# Integration of metabolic and inflammatory mediator profiles as a potential prognostic approach for septic shock in the intensive care unit

**DOI:** 10.1186/s13054-014-0729-0

**Published:** 2015-01-15

**Authors:** Beata Mickiewicz, Patrick Tam, Craig N Jenne, Caroline Leger, Josee Wong, Brent W Winston, Christopher Doig, Paul Kubes, Hans J Vogel, for the Alberta Sepsis Network

**Affiliations:** Bio-NMR-Centre, Department of Biological Sciences, University of Calgary, 2500 University Drive Northwest, Calgary, AB T2N 1N4 Canada; Snyder Translational Laboratory, Department of Critical Care Medicine, University of Calgary, 3280 Hospital Drive Northwest, Calgary, AB T2N 4N1 Canada; Critical Care Epidemiologic and Biologic Tissue Resource, Department of Critical Care Medicine, University of Calgary, 3280 Hospital Drive Northwest, Calgary, AB T2N 4N1 Canada; Department of Community Health Sciences, University of Calgary, 3280 Hospital Drive Northwest, Calgary, AB T2N 4N1 Canada; Snyder Institute for Chronic Diseases, University of Calgary, 3280 Hospital Drive Northwest, Calgary, AB T2N 4N1 Canada

## Abstract

**Introduction:**

Septic shock is a major life-threatening condition in critically ill patients and it is well known that early recognition of septic shock and expedient initiation of appropriate treatment improves patient outcome. Unfortunately, to date no single compound has shown sufficient sensitivity and specificity to be used as a routine biomarker for early diagnosis and prognosis of septic shock in the intensive care unit (ICU). Therefore, the identification of new diagnostic tools remains a priority for increasing the survival rate of ICU patients. In this study, we have evaluated whether a combined nuclear magnetic resonance spectroscopy-based metabolomics and a multiplex cytokine/chemokine profiling approach could be used for diagnosis and prognostic evaluation of septic shock patients in the ICU.

**Methods:**

Serum and plasma samples were collected from septic shock patients and ICU controls (ICU patients with the systemic inflammatory response syndrome but not suspected of having an infection). ^1^H Nuclear magnetic resonance spectra were analyzed and quantified using the targeted profiling methodology. The analysis of the inflammatory mediators was performed using human cytokine and chemokine assay kits.

**Results:**

By using multivariate statistical analysis we were able to distinguish patient groups and detect specific metabolic and cytokine/chemokine patterns associated with septic shock and its mortality. These metabolites and cytokines/chemokines represent candidate biomarkers of the human response to septic shock and have the potential to improve early diagnosis and prognosis of septic shock.

**Conclusions:**

Our findings show that integration of quantitative metabolic and inflammatory mediator data can be utilized for the diagnosis and prognosis of septic shock in the ICU.

**Electronic supplementary material:**

The online version of this article (doi:10.1186/s13054-014-0729-0) contains supplementary material, which is available to authorized users.

## Introduction

Approximately 18 million cases of sepsis occur every year worldwide with a mortality rate reaching almost 30% [1]. However, it has been reported that detecting sepsis, especially at an early stage, improves patient outcome and reduces the mortality rate [2]. Therefore, it is critical to identify new diagnostic tools and develop prognostic approaches to improve patient care and decrease the sepsis death rate.

In recent years, several studies have been performed to describe and identify biomarkers that could be used in the diagnosis and management of sepsis [3]. This previous work has suggested that sepsis could be diagnosed by measuring increased levels of particular proteins in blood such as plasma C-reactive protein, inflammatory cytokines (for example tumor necrosis factor α (TNF-α), interleukin-1 (IL-1) and IL-6), procalcitonin or lipopolysaccharide-binding protein [4]. It has also been reported that the concentrations of lactate or different plasma amino acids can be elevated during the disease [3,5,6]. However, insufficient sensitivity and specificity of the reported compounds currently impede their usage as standard tools for early diagnosis of sepsis [3,7,8]. Therefore, integrated and multifaceted medical approaches supported by effective diagnostic tools, such as a combination of various biomarkers, may improve the identification and the prognosis for sepsis in intensive care units (ICUs) [3,9]. Such an integrated approach, based on merging different data sets, could also create broader and more detailed insight into the nature of the disease than can be achieved using one individual approach.

In this study, we have combined metabolomics and multiplex cytokine/chemokine data to investigate its potential for the diagnosis and prognosis of septic shock. It has previously been demonstrated that nuclear magnetic resonance (NMR) spectroscopy-based metabolomics is a very efficient approach for the discovery of molecular markers of sepsis in animals models [10-12] and within humans [13,14]. In addition, it has been reported that multiplex analysis of cytokines can be used for biomarker identification and quantification in lipopolysaccharide-stimulated human plasma samples [15]. However, only a limited number of studies have demonstrated success in using a multiplex cytokine/chemokine profiling approach for the prediction of sepsis in clinical settings [16-18]. Moreover, to date integration of metabolomics and inflammatory mediator data to identify correlations between immune features and metabolic phenotypes during infection has only been described in an animal model [19]. By using ^1^H NMR spectroscopy and multiplex technology we were able to identify and quantify specific metabolites and inflammatory mediators potentially involved in the septic shock response. Using multivariate statistical analysis we could generate powerful models for diagnosis and prognosis of septic shock. This study presents a promising approach for improving patient care and patient outcome in the ICU and deserves further evaluation in other clinical settings, such as the emergency department.

## Methods

### Sample collection

The study received approval from the Conjoint Health Research Ethics Board of the University of Calgary. The samples were collected in accordance with the guidelines of the Tri-Council policy statement and as part of the Critical Care Epidemiological and Biological Tissue Resource. All patients, or their next of kin, provided written informed consent for participation in this study. The protocol of sample collection has been previously described in detail [14]. Briefly, blood was drawn from patients more than 18 years old that were admitted to the ICU of the Foothills Medical Center or the Peter Lougheed Hospital in Calgary (AB, Canada). The study includes samples collected from septic shock patients who met criteria for septic shock as defined by the American- European consensus statement on sepsis [20,21] and samples obtained from ICU control patients (ICU patients with the systemic inflammatory response syndrome (SIRS) but not suspected of having an infection). All samples were collected within 24 hours of patient admission to the ICU. Serum was obtained by collecting the blood into a sterile silicone-coated vacutainer (BD, Franklin Lakes, NJ, USA) and allowing the blood to clot for 45 min at room temperature. Plasma was obtained by collecting the blood into a sodium-heparin-containing vacutainer (BD) and processed immediately after collection. The samples were then centrifuged at 1200 x g for 15 min, collected into one each 15 mL tube and frozen at −80°C. The samples were thawed once and aliquoted into 250 μL aliquots that were stored at −80°C until used.

### NMR spectroscopy and metabolite concentration profiling

The protocol for the sample preparation and NMR spectral acquisition has been previously described in detail [13,14]. Briefly, filtration (3-kiloDalton (kDa) NanoSep microcentrifuge filters; Pall, Inc., East Hills, NY, USA) of serum samples (V = 250 μL) was followed by adding to the filtrated samples 80 μL of phosphate-buffered solution (0.5 M NaH_2_PO_4_ containing 2.5 mM 2,2-dimethyl-2-silapentane-5-sulfonate, DSS), 10 μL of sodium azide (1 M NaN_3_) and D_2_O. The final volume of each sample was 400 μL and the pH was in the range of 7.0 ± 0.04. NMR spectra were obtained on a 600 MHz Bruker Ultrashield Plus NMR spectrometer (Bruker BioSpin Ltd., Milton, ON, Canada) using a standard Bruker 1D spectroscopy presaturation pulse sequence (noesypr1d) with optimal water suppression and a mixing time of 100 ms [22,23]. The spectra were manually corrected (phasing, baseline correction, referencing to the DSS peak at 0.0 ppm) in the Chenomx NMR Suite 6.1 software (Chenomx Inc., Edmonton, AB, Canada) [23]. The targeted profiling methodology was used for metabolite identification and quantification [23]. If the metabolite peaks could not be distinguished from the noise in NMR spectra, the peaks were assigned with zero value and considered as missing data.

### Cytokine/chemokine profiling

The analysis of the inflammatory mediators in human plasma samples was performed using two human cytokine and chemokine assay kits (Bio-Plex Pro Human Cytokine 21-plex Assay and Bio-Plex Pro Human Cytokine 27-plex Assay), which were obtained from Bio-Rad Laboratories, Inc. (Hercules, CA, USA). Samples were assayed according to the manufacturer’s specifications and the plates were read on a Luminex 200 apparatus (Applied Cytometry Systems, Sheffield, UK). The acquisition and analysis of these samples were performed with Bio-Plex Manager 6.0 (Bio-Rad Laboratories, Inc.). If the coefficient of variance between two replicates was more than 20% the data was considered as a missing value.

### Statistical modeling

The SIMCA-P+ 12.0.1 software (Umetrics, Malmo, Sweden) was applied to create and analyze multivariate models. All metabolites or inflammatory mediators with more than 50% missing values were excluded from the analysis. Data preprocessing (median fold change normalization, logarithmic transformation, centering and unit variance scaling [24]) was first conducted separately for the metabolomics and cytokine/chemokine dataset and then for the combined dataset.

Principal component analysis (PCA) was used to summarize the variation in each data matrix and to show outlying samples, that is samples that are situated outside of the 95% confidence interval of the Hotelling’s T-squared distribution (elliptic or spherical area in the score scatter plots) [25]. Following this, supervised orthogonal partial least squares discriminant analysis (OPLS-DA) was applied [26]. For the integrated metabolomics and cytokine/chemokine data and for the age-sex-matched (age within 5 years) septic shock survivors and nonsurvivors, the OPLS-DA models were based on potentially relevant metabolites selected in two-sample *t* tests with *P* value less than 0.2 as a threshold [27]. To validate the statistical significance of each OPLS-DA model R2Y and Q2 metrics were calculated based on sevenfold cross-validation (CV) [28] (for the mortality model a fourfold CV was used due to the smaller number of samples [25]). The R2Y parameter describes the percentage of variation explained by the model and Q2 describes the predictive ability of the model. The difference between R2Y and Q2 values provides reliable information about the model’s goodness-of-fit and if the difference exceeds 0.2 to 0.3 it indicates an overfitted model and the presence of irrelevant predictors [25].

To reveal the most important metabolites and cytokines/chemokines associated with septic shock and mortality the OPLS-DA regression coefficients were calculated from the input data. The OPLS coefficients were multiplied by the scaling weights for better interpretation [29] and only metabolites and cytokines/chemokines with significant changes in concentration (*P* <0.05) were considered as potential biomarkers.

Additionally, an area under the receiver operating characteristic curve (AUROC) [30] was calculated for each OPLS-DA model (Metz ROC Sofware, The University of Chicago, IL, USA). Specificity, sensitivity and accuracy were determined on the basis of sample class prediction during the cross-validation (Y-predcv, predictive Y variables, in the SIMCA-P+ software). The results of the ROC analysis were then compared to the predictive values of acute physiology and chronic health evaluation (APACHE) II scores [31] and sequential organ failure assessment (SOFA) scores [32] collected for the patients upon admittance to the ICU.

## Results

### Profiled samples

In total 57 samples (37 septic shock patients and 20 ICU controls) were retrospectively selected from the ICU tissue bank for this study. The demographic and clinical characteristics of all the patients enrolled in the study are shown in Table 1. Overall 60 metabolites and 45 cytokines/chemokines were assigned and profiled in the samples. A total of 1.8% missing values was observed in the NMR dataset and a total of 0.7% missing values was detected in the cytokine/chemokine dataset. In both datasets the missing values were randomly distributed. We have recently already presented an analysis of the metabolomics data for a slightly larger cohort [14]. However, the number of samples analyzed here, as well the normalization applied to the NMR data, are distinct from the previous study. The different normalization was required to allow for the subsequent integration of the NMR and multiplex data.

**Table 1:** **Demographic and clinical characteristics of the enrolled patients**

**Characteristic**	**ICU control patients**	**Septic shock patients**
Number of patients	20	37
Males : Females (n)	15 : 5	20 : 17
Age (years)	65.5 (55.5–71)	62 (56–73)
Admission APACHE^*^	14 (12.5–16.5)	23 (16–31)
Admission SOFA	8 (4.5–9)	9 (5.0–12)
Primary ICNARC code (n)	CABG for acute crescendo or unstable angina: 9	Septic shock: 26
	CABG for chronic angina: 4	Bacterial pneumonia: 4
	CABG for acute myocardial infarction: 2	Small bowel infarction due to herniation, volvulus or adhesions: 1
	Spinal stenosis: 2	Cor pulmonale: 1
	Chronic angina: 1	Primary peritonitis: 1
	Traumatic rupture or laceration of spleen: 1	Infective arthritis: 1
	Burns: 1	Inhalation pneumonitis (gastrointestinal contents): 1
		Cystitis, pyocystis or urethritis: 1
		Appendicitis or appendix abscess: 1
Length of ICU stay^*^ (days)	1.6 (1.0–2.5)	5.5 (3.1–9.9)
Patients with organ insufficiency^*^ (n, %)	1 (5%)	11 (30%)
Primary focus of infection (n)	n/a	Lung: 14
		Gynecologic or intra-abdominal: 12
		Catheter related bloodstream infection: 4
		Urinary tract: 3
		Bone/joint: 3
		Head/ears/nose/throat: 1
Confirmed infection (n, %):	n/a	Gram-positive bacteria: 12 (32%)
		Gram-negative bacteria: 12 (32%)
Deaths^*^ (n, %)	0	14 (38%)

^*^Statistically significant feature (*P* <0.05). Primary Intensive Care National Audit and Research Centre (ICNARC) code, acute physiology and chronic health evaluation (APACHE) and sequential organ failure assessment (SOFA) scores were assessed upon admittance to the ICU (intensive care unit). All data are median (interquartile range) unless otherwise noted. CABG, coronary artery bypass surgery.

### Predictive models for metabolomics and cytokine/chemokine data

**Figure 1:**
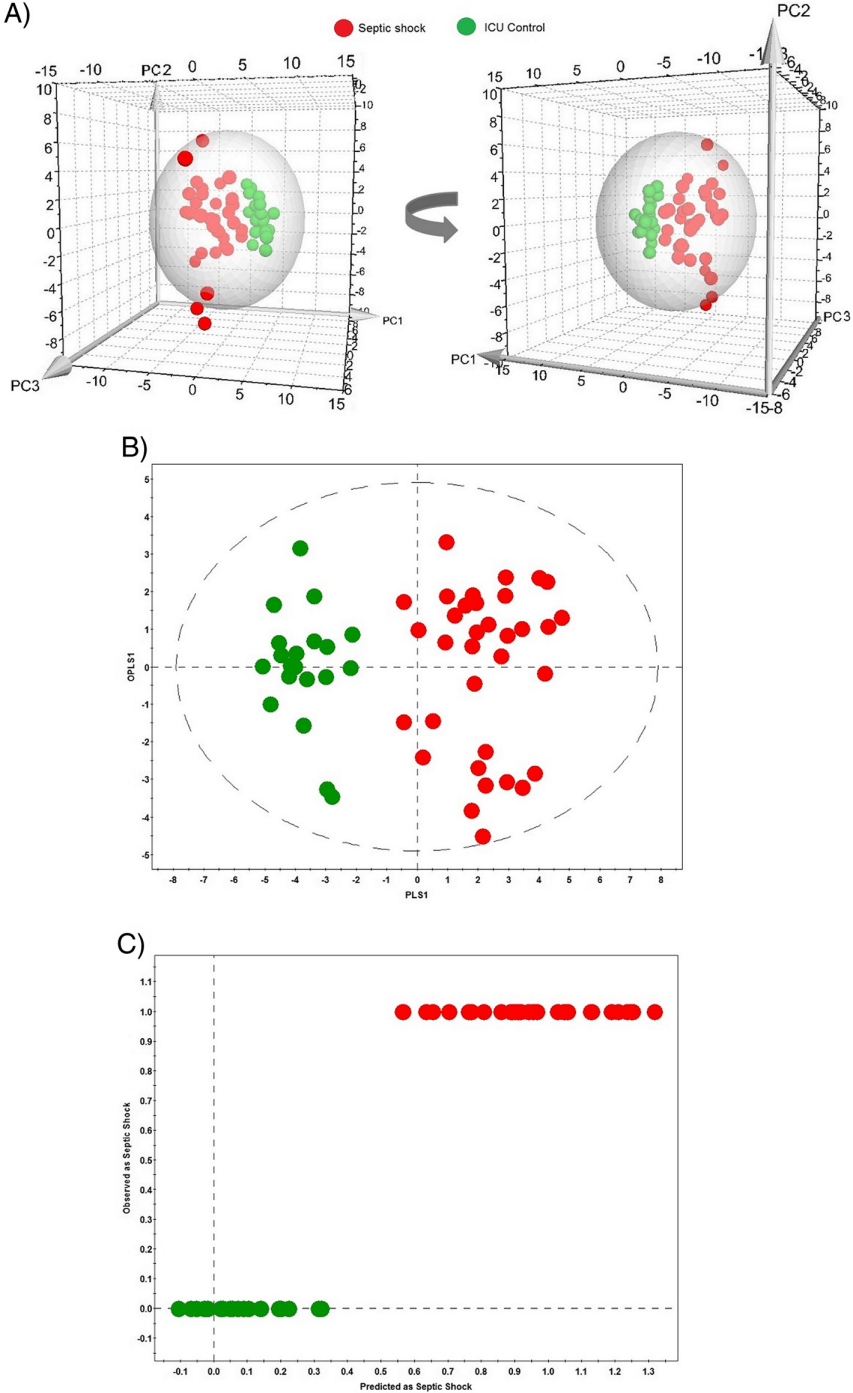
**Septic shock patients versus ICU controls.** Statistical analysis for septic shock patients (red) and ICU controls (green) based on the combined metabolomics and cytokine/chemokine dataset. **(A)** Three-dimensional PCA score scatter plot; **(B)** OPLS-DA score scatter plot; **(C)** ‘Predicted vs. Observed’ plot. The groups are well clustered along the axes of the three principal components in the three-dimensional PCA plot. Three septic shock samples are placed outside the sphere that indicates the 95% confidence interval of the Hotelling’s T-squared distribution. ICU, intensive care unit; OPLS-DA, orthogonal partial least squares discriminant analysis; PCA, principal component analysis.

Figure 1A presents the score scatter plot for the combined dataset. Similar plots for the individual NMR and cytokine/chemokine datasets are shown in Additional file 1. Three principal components (PCs) were calculated to build the PCA models for metabolomics, cytokine/chemokine (Additional file 1) and the combined dataset (Figure 1A).The percentage of variation explained by each component is as follows: for the metabolomics data: PC1 = 13.6%, PC2 = 12.1% and PC3 = 11.5%; for the cytokine/chemokine data: PC1 = 34%, PC2 = 12.4% and PC3 = 6.8%; and for the combined dataset: PC1 = 18.1%, PC2 = 11.1% and PC3 = 9.5%. Some of the septic shock samples (one sample for the metabolomics and cytokine/chemokine model and three samples for the combined dataset) appear far outside of the area of 95% confidence interval of the Hotelling’s T-squared distribution. It is well known that such outliers may disturb the model and incorrectly influence the results [25], thus in the next steps of statistical analysis the data for these samples were excluded. The outlying sample detected in the NMR dataset was exactly the same as in the cytokine/chemokine model. This same outlier was also observed in the combined dataset and from the clinical data this outlying sample was collected from the oldest patient in the whole cohort (88 years old) who was assessed with the admission SOFA score = 0 and who did not survive during the ICU stay.

Next, the supervised OPLS-DA method was applied, including 56 samples for metabolomics and cytokine/chemokine dataset (Additional file 2) and 54 samples for the combined dataset (Figure 1B). The OPLS-DA score scatter plots demonstrate that the samples are very well distinguished, showing the best separation for the combined dataset. The values of R2Y and Q2 metrics are high in all cases with the highest values for the combined dataset (metabolomics data: R2Y = 0.75, Q2 = 0.68; cytokine/chemokine data: R2Y = 0.74, Q2 = 0.66; combined dataset: R2Y = 0.85, Q2 = 0.74). The ‘Predicted vs. Observed’ plot of the combined dataset (Figure 1C) shows that all of the septic shock samples were correctly assigned during the model construction, which indicates a strong predictive ability of the model for septic shock.

**Figure 2:**
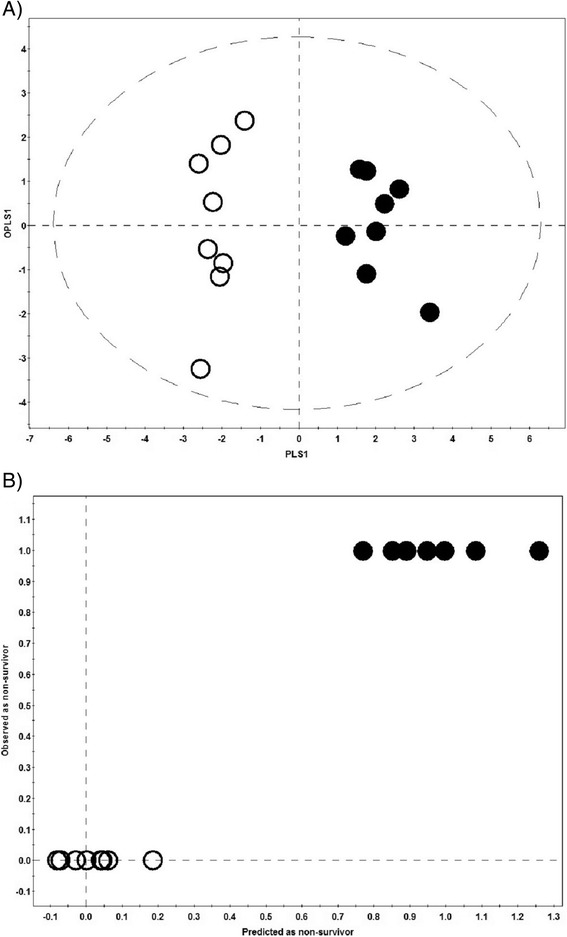
**Mortality model.** The OPLS-DA score scatter plot **(A)** and the ‘Predicted vs. Observed’ plot **(B)** for septic shock nonsurvivors (black dots) and age-sex-matched survivors (black circles) based on the combined metabolomics and cytokine/chemokine dataset. Both groups are well separated along the first PLS component and none of the nonsurvivors were predicted as a survivor. In figure 2B only seven dots are visible instead of eight because two samples had a very similar predicted value and their symbols overlap. OPLS-DA, orthogonal partial least squares discriminant analysis; PLS, partial least squares.

Additionally, we applied OPLS-DA for the prediction of ICU patient outcome. From the available septic shock samples we selected eight nonsurvivors and eight age-sex-matched survivors. The median age of these patients was 63 (59.8 to 77), admission APACHE II score: 25.5 (17.5 to 31.3), admission SOFA score: 10.5 (7 to 12.5) and the length of ICU stay: 6.4 days (3.5 to 9.6 days). The score scatter plot (Figure 2A) and ‘Predicted vs. Observed’ plot (Figure 2B) reveals that septic shock survivors are very well separated from the nonsurvivors. The R2Y, Q2 metric and AUROC have very high values: 0.94, 0.74 and 1.0 respectively. A summary of the quantitative model evaluation results for the various OPLS-DA models that were constructed is presented in Table 2.

**Table 2:** **Statistical analysis results**

**Model**	**Data**	**Sensitivity : Specificity**	**α**	**β**	**PPV : NPV**	**ACC**	**AUROC**
Septic shock vs. ICU controls	Metabolomics	0.92 : 1.0	0	0.08	1.0 : 0.87	0.95	0.99 ± 0.01
	Cytokines/chemokines	0.94 : 0.90	0.1	0.06	0.94 : 0.90	0.93	0.99 ± 0.01
	Combined	0.94 : 1.0	0	0.06	1.0 : 0.91	0.96	1.0
	APACHE	0.82 : 0.42	0.58	0.18	0.71 : 0.57	0.67	0.74 ± 0.07
	SOFA	0.85 : 0.25	0.75	0.15	0.66 : 0.50	0.63	0.64 ± 0.07
Nonsurvivors vs. survivors	Combined	1.0 : 0.88	0.13	0	0.89 : 1.0	0.94	1.0
	APACHE	0.63 : 0.75	0.25	0.38	0.71 : 0.67	0.69	0.78 ± 0.12
	SOFA	0.75 : 0.63	0.38	0.25	0.67 : 0.71	0.69	0.81 ± 0.11

Comparison of statistical measures for septic shock patients vs. ICU controls and septic shock nonsurvivors vs. septic shock survivors models based on metabolomics data, cytokine/chemokine data, combined dataset (metabolites together with inflammatory mediators), acute physiology and chronic health evaluation (APACHE) and sequential organ failure assessment (SOFA) scores. The receiver operating characteristic (ROC) curve plots for each dataset are shown in Figure 4. α, false positive rate; β, false negative rate; PPV, positive predictive value; NPV, negative predictive value; ACC, accuracy; AUROC, area under the receiver operating characteristic curve (value ± standard error as calculated from the ROC curves); ICU, intensive care unit.

Following a suggestion of an anonymous reviewer of this work we also recalculated the supervised models including the outliers identified in the PCA. This only had a minor influence on the models (see Additional file 3).

### Biomarkers

**Figure 3:**
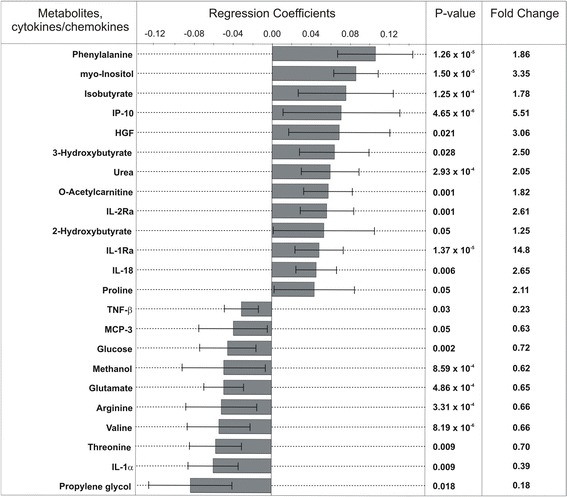
**The OPLS-DA regression coefficient plot.** Positive values of coefficients (the upper part of the diagram) indicate increased metabolite and cytokine/chemokine concentrations in the septic shock samples (fold change >1) while negative values (the lower part of diagram) present a decrease in metabolite and cytokine/chemokine concentrations, as compared to ICU controls (fold change <1). Only significant metabolites and cytokines/chemokines are shown (*P* <0.05, two-sample *t* test). ICU, intensive care unit; OPLS-DA, orthogonal partial least squares discriminant analysis.

As shown in Figure 3, fifteen metabolites and eight inflammatory mediators contribute significantly to the separation between septic shock samples and ICU controls. Eight metabolites revealed altered concentrations in septic shock patients (phenylalanine, myo-inositol, isobutyrate, 3-hydroxybutyrate, urea, O-acetylcarnitine, 2-hydroxybutyrate and proline) while the concentrations of propylene glycol, threonine, valine, arginine, glutamate, methanol and glucose were decreased. Septic shock patients showed also high levels of interferon-inducible protein-10 (IP-10), hepatocyte growth factor (HGF), interleukin-18 (IL-18), IL-1 and IL-2 receptor antagonists (IL-1Ra, IL-2Ra) and decreased concentrations of IL-1α, monocyte-specific chemokine 3 (MCP-3) and tumor necrosis factor beta (TNF-β).

Additionally, we were able to reveal metabolic and cytokine/chemokine factors associated with septic shock mortality (Additional file 4). Elevated levels of 2-hydroxyisovalerate, fructose, IL-8, IL-9 and growth regulated oncogene alpha (GRO-α) and decreased concentrations of TNF-β, beta-nerve growth factor (β-NGF) and dimethylamine were detected in septic shock nonsurvivors compared to the survivors.

### Comparison with ICU scoring systems

**Figure 4:**
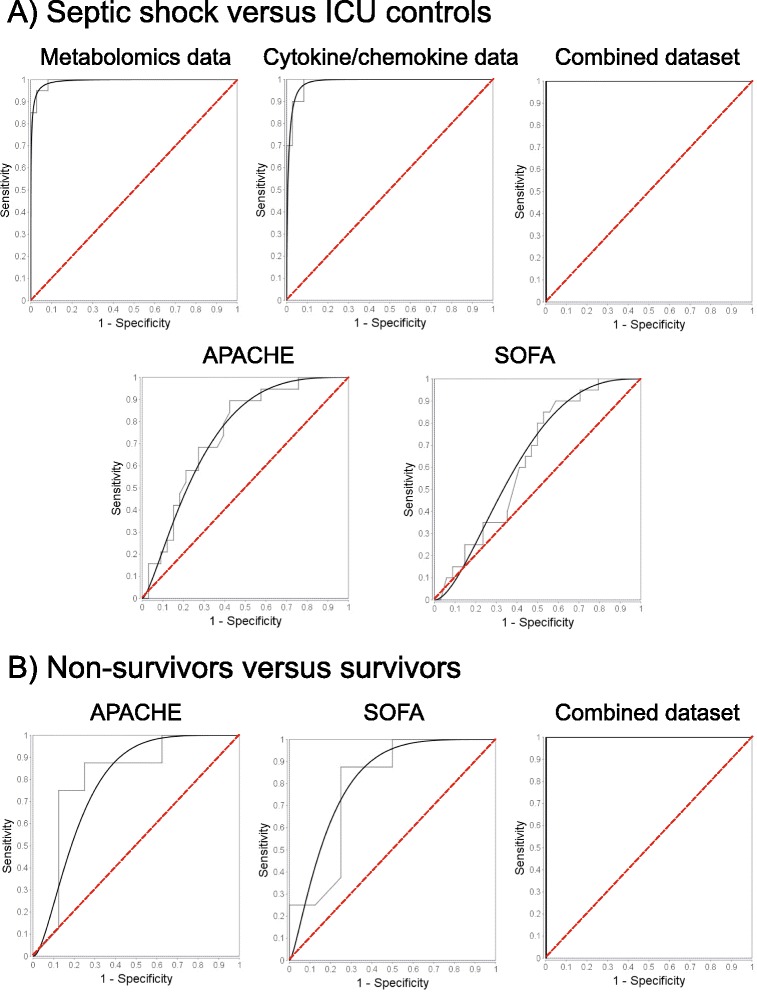
**The receiver operating characteristic (ROC) curve plots.** The ROC plots for **(A)** septic shock patients vs. intensive care unit (ICU) controls and **(B)** septic shock nonsurvivors vs. septic shock survivors models based on the metabolomics data, cytokine/chemokine data and the combined dataset (metabolites together with inflammatory mediators), acute physiology and chronic health evaluation (APACHE) and sequential organ failure assessment (SOFA) scores. Black line - fit line, grey line - empirical data, red dashed line - the chance curve. To further show the details of these curves in the range of false positive fraction the Additional file 5 shows the ROC curves redrawn with a decimal logarithm scale for the horizontal axes.

In order to provide an indication of the potential clinical usefulness of our approach we made a comparison to commonly used clinical scoring evaluations. The results of ROC analysis indicate the best sensitivity, specificity, accuracy and the highest AUROC values for the integrated metabolomics-cytokine/chemokine approach compared to the diagnostic and prognostic power of APACHE II and SOFA scores (Table 2, Figure 4 and Additional file 5).

## Discussion

The present study is focused on utilizing a combined NMR-based metabolomics and multiplex cytokine/chemokine profiling approach as a potential prognostic evaluation of septic shock. These techniques have several unique advantages over other diagnostic tools. First, quantitative NMR-based metabolomics and bead-based multiplexing for cytokine/chemokine analysis allows for the quantitative measurement of more than 100 different biomarkers from a sample volume less than 250 μL. The ability to maximize this minimal sample volume is essential, as obtaining large samples from critically ill patients, particularly pediatric patients can be difficult. Second, these techniques detect even very small changes in analyte concentration, allowing for the identification of even subtle variations in the patient’s biopattern. Finally, through the analysis of more than 100 biomarkers, we have been able to identify several patterns that otherwise would not have been observed if a smaller, more finite, screen of previously identified biomarkers had been used.

Through the use of multivariate statistical analysis we identified a specific biopattern associated with an early recognition of septic shock. We detected elevated levels of eight metabolites and five inflammatory mediators. Increased concentrations of isobutyrate, myo-inositol, proline and urea indicate hepatic failure and kidney injury [33-36]. 3-hydroxybutyrate, O-acetylcarnitine and 2-hydroxybutyrate are metabolites which correlate to energy demands resulting from rising metabolic requirements and inflammatory responses associated with disease conditions [36,37]. An elevated level of phenylalanine is the result of an accelerated rate of protein breakdown, as often caused by infections and inflammatory states [38].

Additionally, the septic shock patients exhibited a remarkable increase in the levels of IP-10 and HGF. It has already been reported that the concentration of IP-10 was elevated in plasma samples of septic shock patients compared to SIRS patients and that IP-10 might serve as a diagnostic marker [39]. Moreover, it was found that the HGF level was significantly higher in sepsis patients than in the SIRS groups without infection [40], which correlates with our results. We also observed higher levels of IL-18, IL-1Ra and IL-2Ra in septic shock patients as compared to ICU control patients. The proinflammatory cytokine IL-18 has already been characterized as an important regulator of the innate and acquired immune responses [41]. Interestingly, IL-1Ra and IL-2Ra are not proinflammatory molecules *per se*, but instead represent the body’s response to severe inflammation. IL-1Ra is a cytokine and is an IL-1 receptor antagonist, which has been demonstrated to block the proinflammatory activities of IL-1α and IL1-β [42,43]. In contrast, IL-2Ra represents a soluble form of the IL-2 receptor alpha chain that has been released from cell surfaces through the extracellular proteolysis of the IL-2 receptor and functions to bind and block IL-2 resulting in diminished IL-2 signaling [44]. Although these molecules are not proinflammatory themselves, they have been associated with a number of inflammatory diseases and sepsis [45,46] and as such have been proposed to be markers of inflammation.

Furthermore, the concentration of seven metabolites and three inflammatory mediators significantly decreased in septic shock samples compared to the ICU controls. Low levels of glucose and propylene glycol probably result from the rapid oxidation of these metabolites into pyruvate and reflect increasing energy demands during septic shock. The decreased concentration of other compounds (threonine, valine, arginine, glutamate, methanol) is primarily associated with organ dysfunction and/or higher utilization of these metabolites in the disease conditions [47-50]. The low level of IL-1α seems to be directly related to the increased concentration of IL-1Ra detected in our study. Interestingly, the level of TNF-β which dropped in septic shock samples was also decreased in septic shock nonsurvivors compared to the survivors in the mortality model. Therefore, a high concentration of TNF-β in serum sample might indicate lower morbidity and better outcome for the ICU patient. The meaning of the decreased level of MCP-3 in septic shock patients could not be explained as it is not well understood how this chemokine is implicated in septic shock. Clearly, further studies of MCP-3 are needed to confirm its importance.

Nonetheless, many of the metabolites and cytokines/chemokines we have observed to be statistically different between septic shock patients and ICU control patients have been previously identified as molecules of interest in sepsis [3]. Furthermore, many of these molecules have been tested as possible point-of-care diagnostic markers in sepsis but none of the identified markers alone have been adapted into a successful diagnostic test for sepsis [3]. This failure is likely the result of the multifaceted nature of sepsis; a marker that demonstrates significant association with one group of septic patients may not correlate with all septic patients. As a result, the use of single biomarkers in diagnosis of sepsis has not, and likely will not, result in the development of successful point-of-care testing.

It has previously been demonstrated that applying metabolomics or multiple cytokine assays separately allows for an identification of specific markers associated with sepsis severity. However, to date only metabolomics studies of sepsis have described potentially predictive values. The multiplex inflammatory mediators studies did not propose any predictive model that might be used for early diagnosis of septic shock [16]. Recently, another study has assessed a multiple cytokine profiling approach to distinguish SIRS and various forms of sepsis within a group of emergency department patients [17]. Indeed, in this study the authors were able to describe individual mediators independently associated with septic shock. However, the global statistical analysis could not identify any significant differences between the patient groups. In light of these previous reports, our integrated metabolite and cytokine/chemokine study can represent a potentially promising methodology for the prediction of septic shock. The combination of biomarkers such as metabolites and inflammatory mediators yields better results and predictive values than studies previously published and models constructed based on separate datasets only (Table 2).

Additionally, we were able to construct a model for mortality prediction which represents a much better prognostic ability than the commonly used APACHE II and SOFA scores. It should be noted that the application of multiple cytokine assays to predict septic shock outcome has already been described [18]. However, these authors could only observe a significant mortality odds ratio when using the cytokine/chemokine data collected more than 24 hours after patient enrollment. In contrast, our results are based on blood samples obtained at an earlier stage of patient admission to the ICU (not more than 24 hours). It is well known that the first hours following patient diagnosis are the most important for patient survival and prognosis of patient outcome at this time is very crucial. A similar approach has been described in a recent study in which the authors attempted to integrate metabolomics, proteomics and clinical variables to predict the survival of adult sepsis patients [51]. Although they performed a broad proteomics analysis by mass spectrometry, they concluded that these results were at best semi-quantitative and they could not incorporate them in their predictive model. Moreover, they also mentioned that their proteome analysis was not sensitive enough to reliably measure cytokines/chemokines in their samples. Since cytokines are known to play an important role during sepsis, we have used a targeted and quantitative cytokine/chemokine proteomics multiplex approach in this study. Our data clearly illustrate that it is possible to integrate quantitative metabolic and cytokine/chemokine proteomic data in a bigger biomarker panel. Furthermore, our study describes a mortality model that is only based on integrated bio-fluid components. This approach may be advantageous to avoid a possible bias associated with a subjective diagnosis by critical care staff. Be that as it may, our method can also easily be extended to include quantitative clinical variables and severity scores.

## Conclusions

This study indicates that an integrated metabolic and cytokine/chemokine profiling approach of blood samples might serve as a promising tool for the early diagnosis and prognosis of septic shock during the first hours of patient admission to the ICU. However, this study should be considered as an initial step of applying integrated metabolomics and inflammatory mediator profiling approach in a clinical setting. Beyond doubt, our results should be validated in other clinical settings and within larger groups of patients to confirm its applicability throughout different ICUs. Additionally, correlation of metabolic and cytokine/chemokine profiles with the severity of sepsis, as well as validation of the model in an early sepsis patient population, would provide further insight into disease mechanisms and could be used to target new therapies in the future.

## Key messages

- Integration of metabolic and inflammatory mediator profiling data might serve as a reliable diagnostic and prognostic tool for septic shock.
- A total of fifteen metabolites and eight inflammatory mediators had a significant influence on the separation between septic shock samples and ICU controls.
- A receiver operating characteristic analysis indicated an excellent predictive ability of the integrated metabolomics/inflammatory mediator models when compared to the conventionally used ICU scoring systems.

## Additional files

Additional file 1:
**Three-dimensional PCA score scatter plots.** Three-dimensional PCA score scatter plots obtained for 37 septic shock patients (red) and 20 ICU controls (green) based on the (A) metabolic data and (B) cytokine/chemokine data. The groups are well clustered along the axes of the three principal components. One septic shock sample is placed outside the sphere that describes the 95% confidence interval of the Hotelling’s T-squared distribution. Additional file 2:
**OPLS-DA score scatter plots.** The OPLS-DA score scatter plots obtained for 36 septic shock patients (red) and 20 ICU controls (green) based on the (A) metabolic data and (B) cytokine/chemokine data. Additional file 3:
**The summary table.** Comparison of statistical measures calculated for the supervised OPLS-DA models without excluding outliers to the results presented in the manuscript. Additional file 4:
**The OPLS-DA regression coefficient plot.** Positive values of coefficients (the upper part of the diagram) indicates increased metabolite or inflammatory mediator concentrations in septic shock nonsurvivor samples while negative values (the lower part of diagram) present a decrease in metabolite or inflammatory mediator concentrations, as compared to the age-sex-matched septic shock survivors. Only significant metabolites are shown (*P* <0.05, two-sample *t* test). Additional file 5:
**The receiver operating characteristic (ROC) curves plotted with a decimal logarithmic scale for the horizontal axes.** The ROC plots for (A) septic shock patients vs. ICU controls and (B) septic shock nonsurvivors vs. septic shock survivors models based on the metabolomics data, cytokine/chemokine data and the combined dataset (metabolites together with inflammatory mediators), APACHE (acute physiology and chronic health evaluation) and SOFA (sequential organ failure assessment) scores. Black line - fit line, red dashed line - the chance curve (that is the diagonal of the ROC curve when plotted in linear scale).

## Abbreviations

ACC: accuracy
APACHE: acute physiology and chronic health evaluation
AUROC: area under the receiver operating characteristic curve
CABG: coronary artery bypass surgery
CV: cross-validation
GRO-α: growth-regulated oncogene alpha
HGF: hepatocyte growth factor
ICNARC: Intensive Care National Audit and Research Centre
ICU: intensive care unit
IL-1: interleukin 1
IL-2: interleukin 2
IL-18: interleukin-18
IL-1Ra: interleukin-1 receptor antagonist
IL-1α: interleukin-1α
IL-2Ra: interleukin-2 receptor antagonist
IL-8: interleukin-8
IL-9: interleukin-9
IP-10: interferon-inducible protein-10
kDa: kiloDaltons
MCP-3: monocyte-specific chemokine 3
NMR: nuclear magnetic resonance
NPV: negative predictive value
OPLS-DA: orthogonal partial least squares discriminant analysis
PC: principal component
PCA: principal component analysis
PLS: partial least squares
PPV: positive predictive value
SIRS: systemic inflammatory response syndrome
SOFA: sequential organ failure assessment
TNF-α: tumor necrosis factor alpha
TNF-β: tumor necrosis factor beta
β-NGF: beta nerve growth factor
