# Supplementary figures and images for: Integration of metabolic and inflammatory mediator profiles as a potential prognostic approach for septic shock in the intensive care unit
Source: Crit Care. 2015 Jan 15;19(1):11. doi: 10.1186/s13054-014-0729-0 (PMC4340832; doi:10.1186/s13054-014-0729-0)

## A) Metabolomics dataset

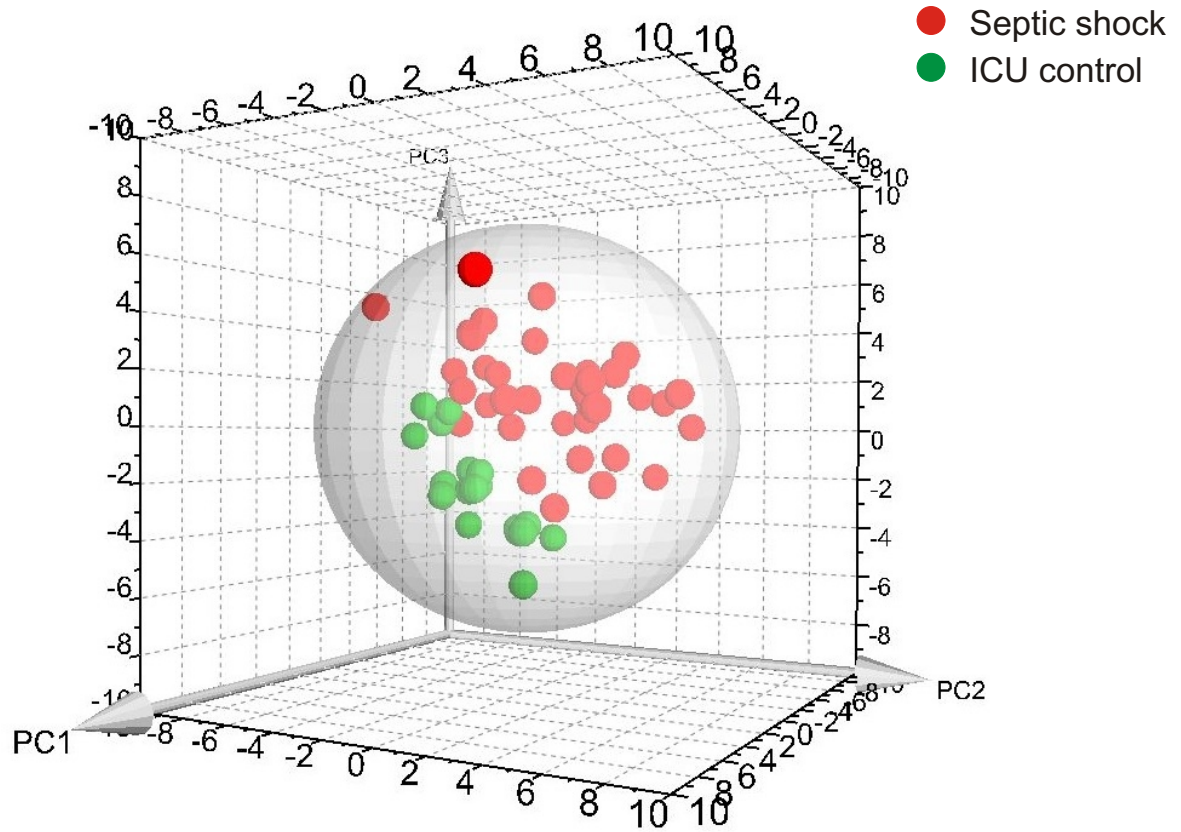

## B) Cytokine/chemokine dataset

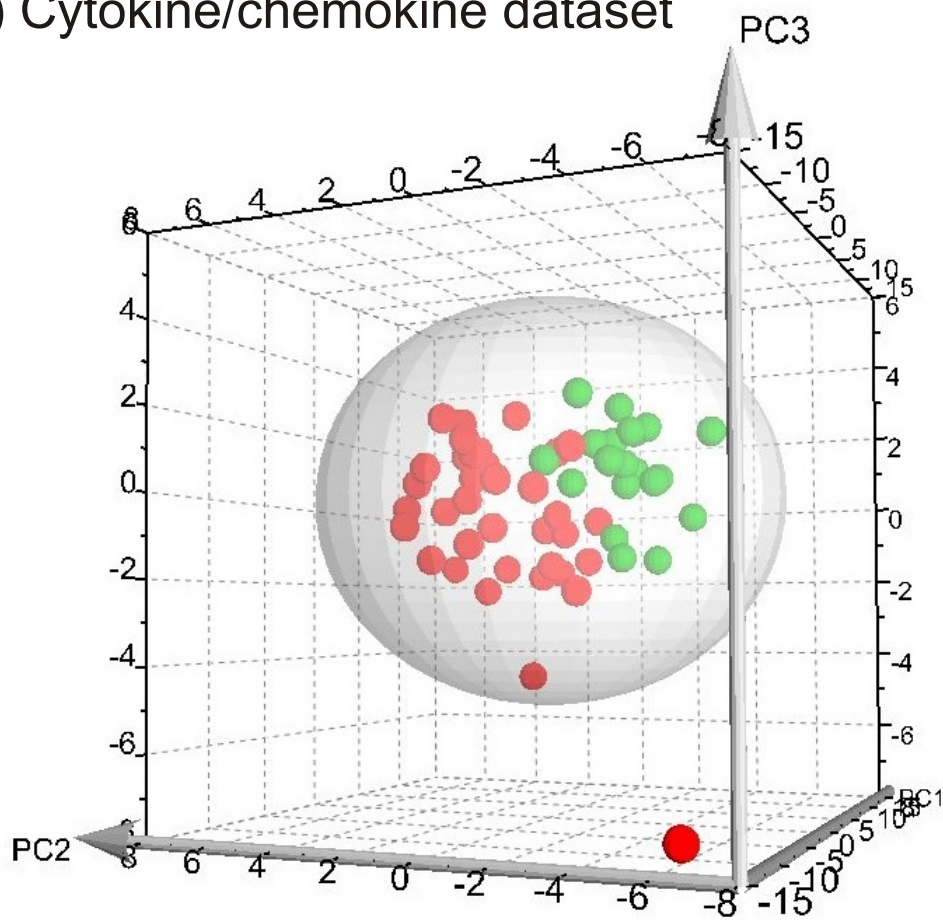

Supplement: Additional file 1: — Three-dimensional PCA score scatter plots. Three-dimensional PCA score scatter plots obtained for 37 septic shock patients (red) and 20 ICU controls (green) based on the (A) metabolic data and (B) cytokine/chemokine data. The groups are well clustered along the axes of the three principal components. One septic shock sample is placed outside the sphere that describes the 95% confidence interval of the Hotelling’s T-squared distribution. [file 13054_2014_729_MOESM1_ESM.pdf]

## A) Metabolomics dataset

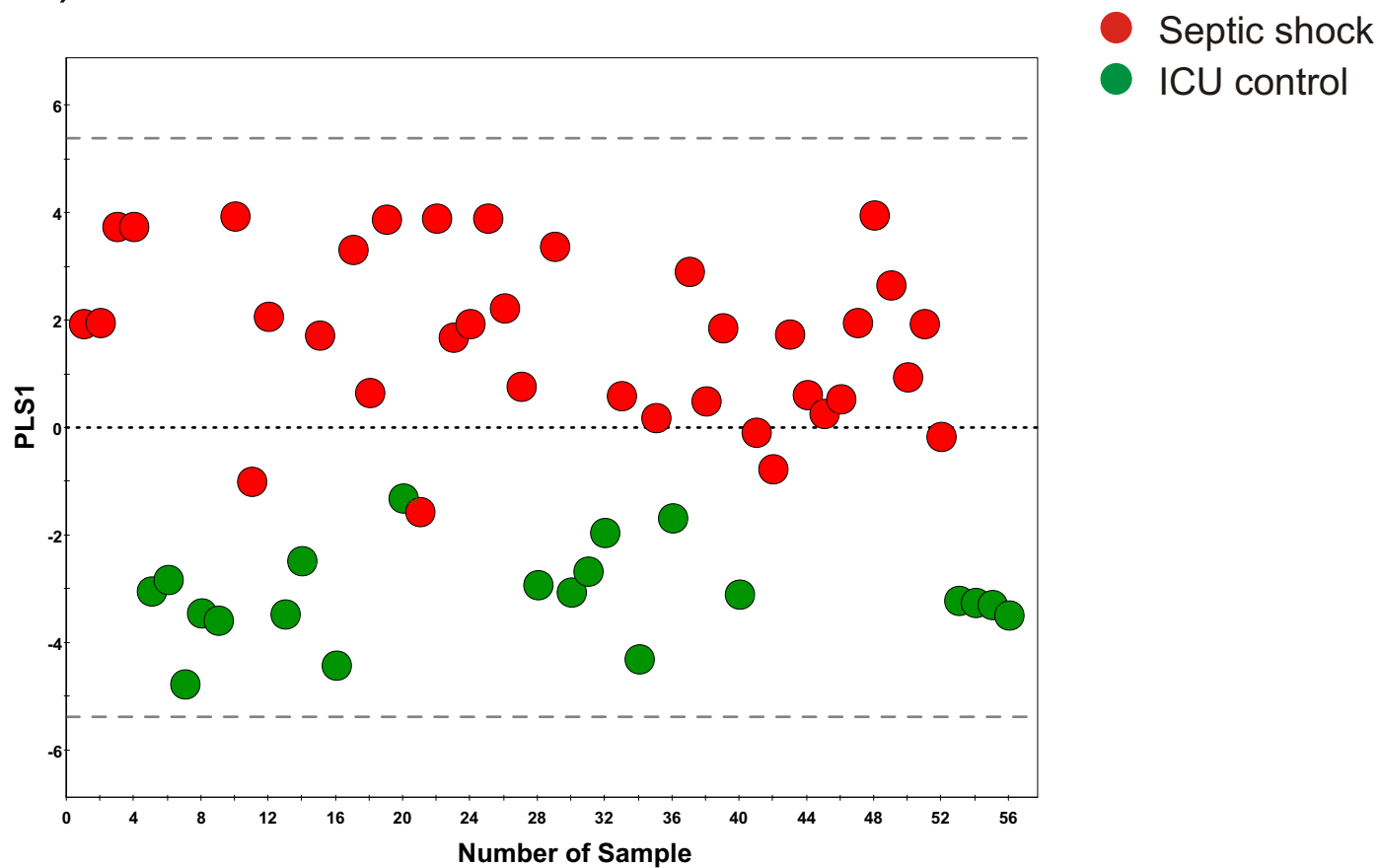

## B) Cytokine/chemokine dataset

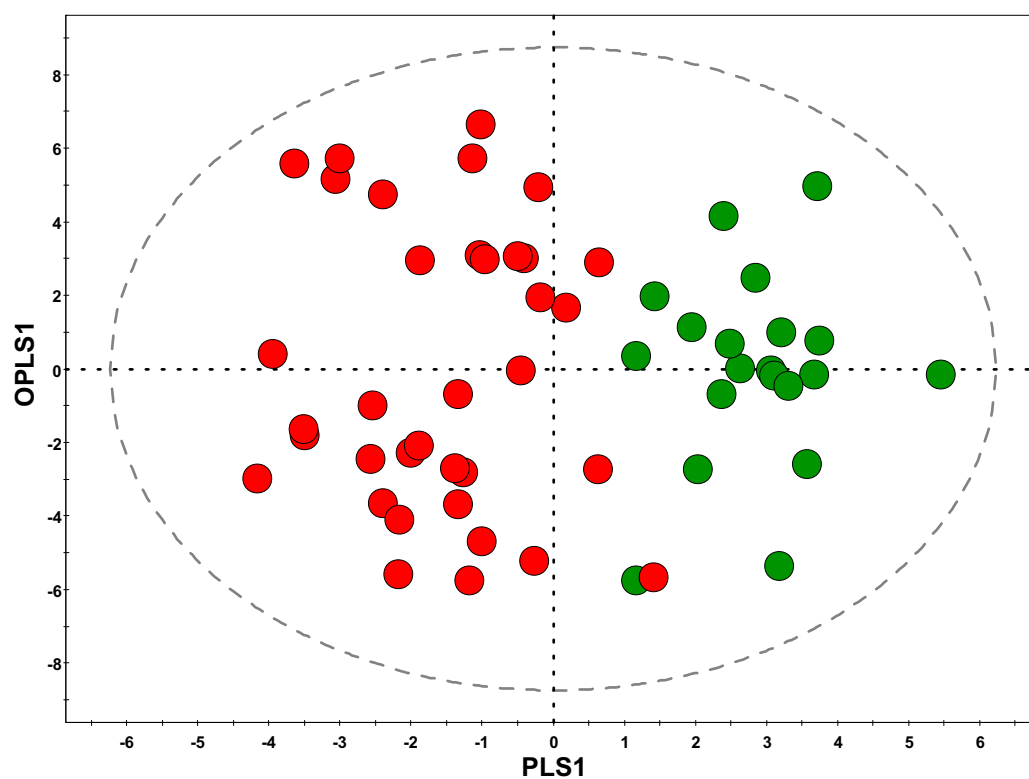

Supplement: Additional file 2: — OPLS-DA score scatter plots. The OPLS-DA score scatter plots obtained for 36 septic shock patients (red) and 20 ICU controls (green) based on the (A) metabolic data and (B) cytokine/chemokine data. [file 13054_2014_729_MOESM2_ESM.pdf]
