# Supplementary material for: Integration of metabolic and inflammatory mediator profiles as a potential prognostic approach for septic shock in the intensive care unit
Source: Crit Care. 2015 Jan 15;19(1):11. doi: 10.1186/s13054-014-0729-0 (PMC4340832; doi:10.1186/s13054-014-0729-0)
Supplement: Additional file 3: — The summary table. Comparison of statistical measures calculated for the supervised OPLS-DA models without excluding outliers to the results presented in the manuscript. [file 13054_2014_729_MOESM3_ESM.pdf]

OPLS-DA model	Outliers included	Outliers excluded (see manuscript)
Metabolomics data	R2Y = 0.76; Q2 = 0.68	R2Y = 0.75; Q2 = 0.68
Cytokine/chemokine data	R2Y = 0.72; Q2 = 0.61	R2Y = 0.74; Q2 = 0.66
Combined dataset	R2Y = 0.85; Q2 = 0.72	R2Y = 0.85; Q2 = 0.74
