# Supplementary material for: Integration of metabolic and inflammatory mediator profiles as a potential prognostic approach for septic shock in the intensive care unit
Source: Crit Care. 2015 Jan 15;19(1):11. doi: 10.1186/s13054-014-0729-0 (PMC4340832; doi:10.1186/s13054-014-0729-0)
Supplement: Additional file 4: — The OPLS-DA regression coefficient plot. Positive values of coefficients (the upper part of the diagram) indicates increased metabolite or inflammatory mediator concentrations in septic shock nonsurvivor samples while negative values (the lower part of diagram) present a decrease in metabolite or inflammatory mediator concentrations, as compared to the age-sex-matched septic shock survivors. Only significant metabolites are shown (P <0.05, two-sample t test). [file 13054_2014_729_MOESM4_ESM.pdf]

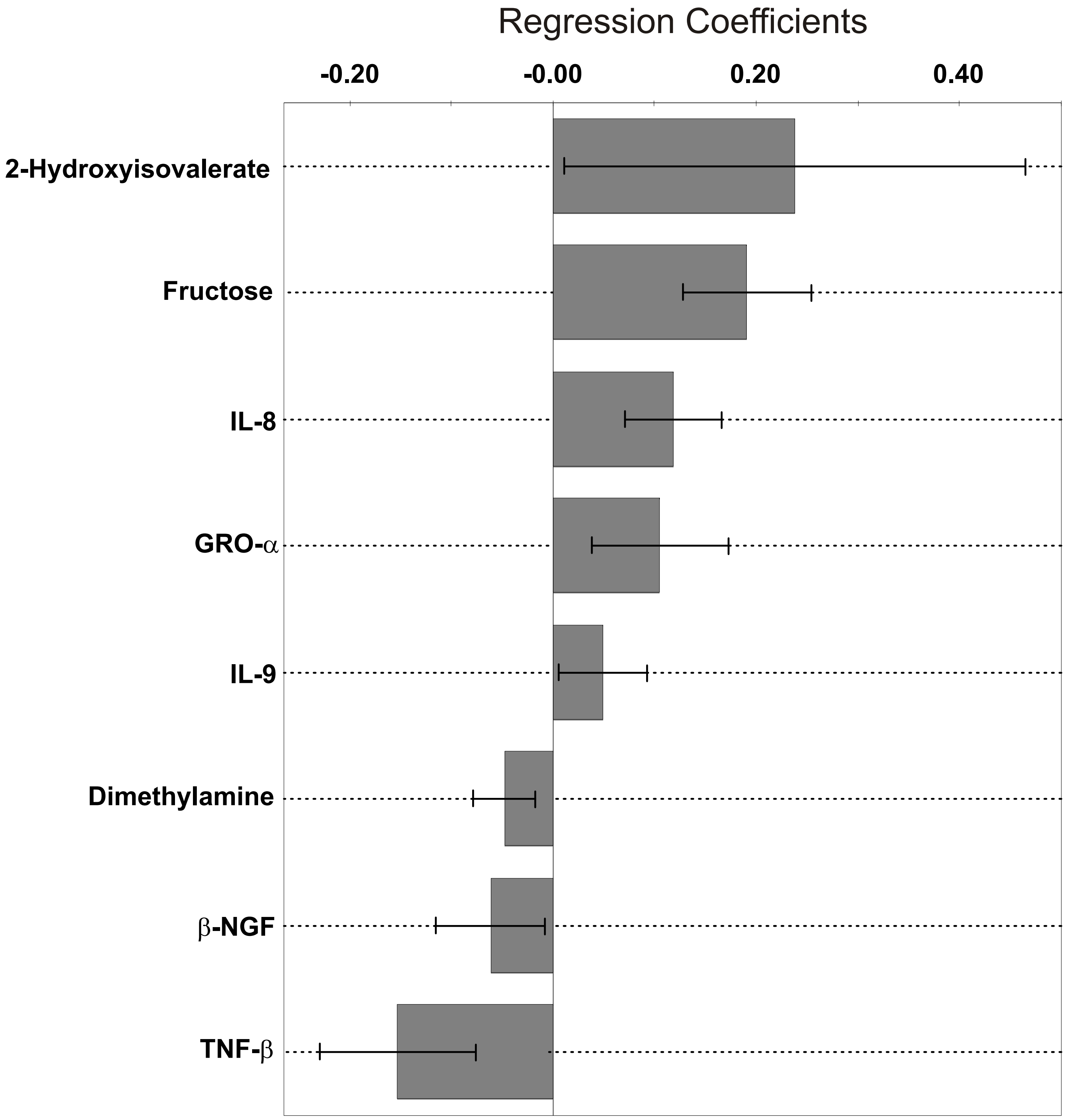
Regression Coefficients
-0.20
-0.00
0.20
0.40
2-Hydroxyisovalerate
Fructose
IL-8
GRO-α
IL-9
Dimethylamine
β-NGF
TNF-β
