# Supplementary material for: Integration of metabolic and inflammatory mediator profiles as a potential prognostic approach for septic shock in the intensive care unit
Source: Crit Care. 2015 Jan 15;19(1):11. doi: 10.1186/s13054-014-0729-0 (PMC4340832; doi:10.1186/s13054-014-0729-0)
Supplement: Additional file 5: — The receiver operating characteristic (ROC) curves plotted with a decimal logarithmic scale for the horizontal axes. The ROC plots for (A) septic shock patients vs. ICU controls and (B) septic shock nonsurvivors vs. septic shock survivors models based on the metabolomics data, cytokine/chemokine data and the combined dataset (metabolites together with inflammatory mediators), APACHE (acute physiology and chronic health evaluation) and SOFA (sequential organ failure assessment) scores. Black line - fit line, red dashed line - the chance curve (that is the diagonal of the ROC curve when plotted in linear scale). [file 13054_2014_729_MOESM5_ESM.pdf]

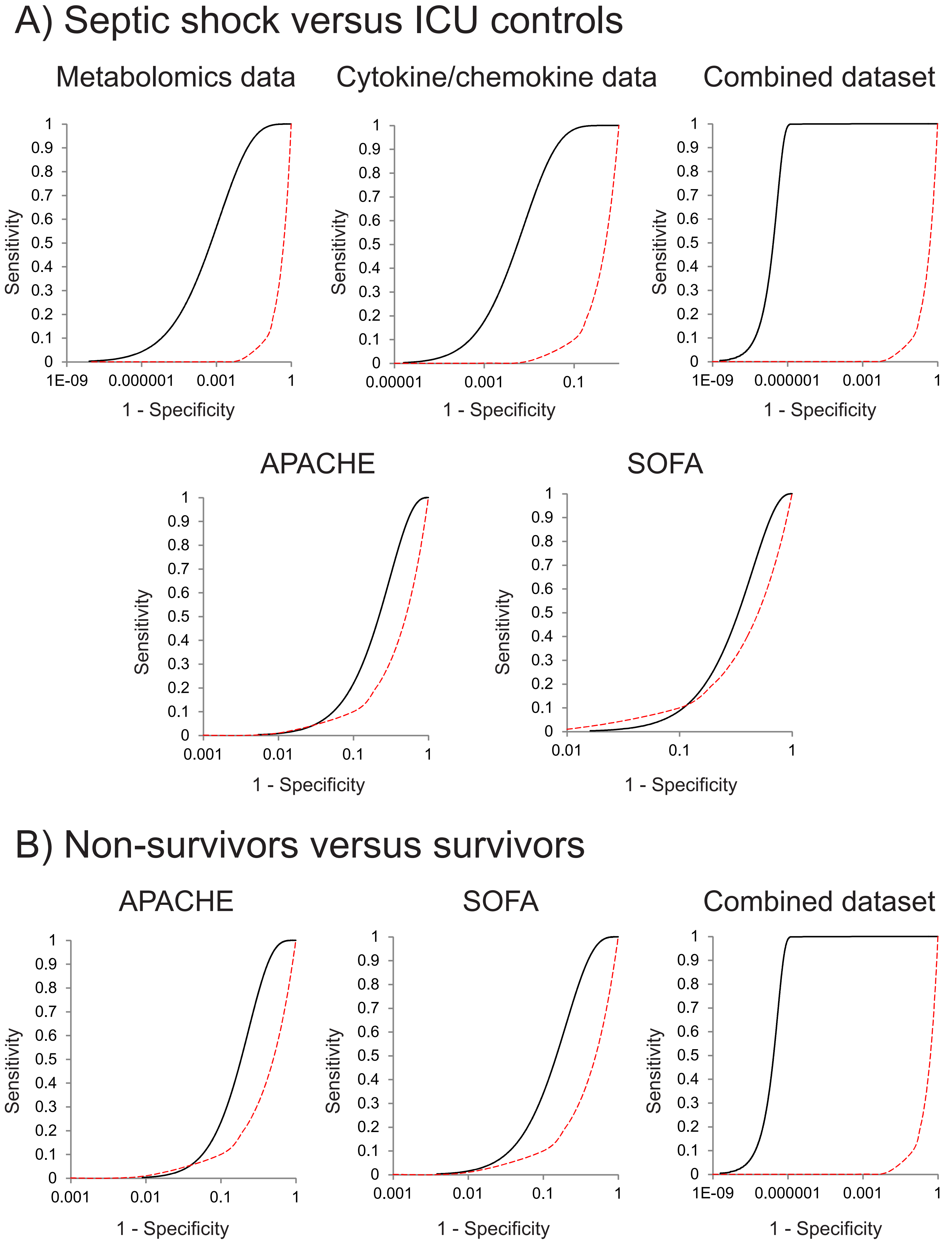
A) Septic shock versus ICU controls
Metabolomics data
Cytokine/chemokine data
Combined dataset
APACHE
SOFA
B) Non-survivors versus survivors
APACHE
SOFA
Combined dataset
Sensitivity
1 - Specificity
0
0.1
0.2
0.3
0.4
0.5
0.6
0.7
0.8
0.9
1
1E-09
0.000001
0.001
0.00001
0.01
0.1
